# Efficacy of fitting HydroCone (Toris K) contact lenses for the visual rehabilitation of patients with posterior microphthalmos

**DOI:** 10.5935/0004-2749.20230051

**Published:** 2023

**Authors:** Gamze Ozturk Karabulut, Ferah Ozcelik

**Affiliations:** 1 Beyoğlu Eye Research and Training Hospital, University of Health Sciences, Istanbul, Turkey.

**Keywords:** Microphthalmos, Contact lens, hydrophilic, Silicone, Vision disorder/rehabilitation, Visual acuity, Microftalmia, Lentes de contato hidrofílicas, Silicones, Transtornos da visão/reabilitação, Acuidade visual

## Abstract

**Purpose:**

To evaluate the efficacy of soft HydroCone silicone hydrogel contact lenses in patients with posterior microphthalmos.

**Methods:**

The charts of 13 patients with posterior microphthalmos (26 eyes) who were fitted with soft HydroCone silicone hydrogel contact lenses were reviewed retrospectively. All the patients underwent assessments of uncorrected and best spectacle-corrected visual acuity and cycloplegic refraction. They were fitted with contact lenses according to the parameter values obtained from the topographical analysis and best contact lens-corrected visual acuity measurement.

**Results:**

The spherical equivalents of the right and left eyes ranged from 10.00 to 19.25 diopters and from 11.00 to 21.5 diopters, respectively. The mean axial and posterior chamber lengths were shorter than those of the age-matched population. However, the mean values of the anterior segment parameters such as horizontal visible iris diameter, central anterior chamber depth, lens thickness, and central corneal thickness were in the normal range. The mean keratometric measurements revealed increased corneal curvature compared with that in the normal population. The mean best contact lens-corrected visual acuity was significantly higher than the mean best spectacle-corrected visual acuity for both eyes (p=0.045).

**Conclusion:**

HydroCone silicon soft contact lenses provided better visual acuity than spectacles in the patients with posterior microphthalmos in this study.

## Abstract (pt)

**Objetivo:**

Avaliar a eficácia das lentes de contato gelatinosas HydroCone, de hidrogel com silicone, em pacientes com microftalmia posterior.

**Métodos:**

Foram revisados retrospectivamente 26 olhos com microftalmia posterior, a partir dos prontuários de 13 pacientes que receberam lentes de contato gelatinosas HydroCone, de hidrogel com silicone. Todos os pacientes foram submetidos ao exame de acuidade visual não corrigida e com melhor correção por óculos e com refração cicloplégica. Todos os pacientes receberam lentes de contato de acordo com os parâmetros obtidos na análise topográfica e foi obtida a melhor acuidade visual corrigida com lentes de contato.

**Resultados:**

O equivalente esférico do olho direito variou de 10,00 a 19,25 dioptrias, e o do olho esquerdo de 11,00 a 21,5 dioptrias. Os comprimentos médios axiais e das câmaras posteriores foram menores do que para a população de mesma idade. No entanto, os valores médios dos parâmetros do segmento anterior, como o diâmetro horizontal visível da íris, a profundidade da câmara anterior central, a espessura da lente e a espessura central da córnea estavam dentro da faixa normal. Os valores médios da ceratometria revelaram curvatura corneana aumentada em relação à população normal. A média da melhor acuidade visual corrigida com lentes de contato foi significativamente maior que a média da melhor acuidade visual corrigida com óculos em ambos os olhos (p=0,045).

**Conclusão:**

As lentes de contato gelatinosas de silicone HydroCone proporcionam melhor acuidade visual que óculos em pacientes com microftalmia posterior.

## INTRODUCTION

Microphthalmos is a disorder where the total axial length of an eye is smaller than 2 standard deviations of the normal length for that age group^(1)^. It is a rare congenital spectrum of diseases, including simple microphthalmos without any other ocular malformations and complex microphthalmos, which is associated with multiple associated ocular and/or systemic abnormalities^(1)^. Posterior microphthalmos (PM) is a rare subgroup of microphthalmos that is usually unrecognized owing to the unremarkable anterior segment of the eye with normal dimensions; however, the posterior segment is disproportionately smaller than normal, which leads to short axial length and high hyperopia^(2,3)^. It is associated with posterior segment abnormalities such as papillomacular folds involving the sensorial retina only^(4-13)^, crowded optic disk^(6,7,13)^, sclerochoroidal thickening^(11)^, uveal effusions^(11)^, absence or reduction of foveal capillary zone^(5)^, pigmentary retinopathy^(14)^, foveaschisis^(14)^, and macular hole^(15)^. Disparity due to the arrested development of the retina pigment epithelium, choroid, and sclera with normal growth of the anterior segment and sensorial retina is the most accepted theory for the development PM^(4,5,12,13)^. In patients with PM, biometric analyses revealed increased corneal curvature with normal dimensions of the anterior segment^(4,7,9,11,16,17)^.

Soft HydroCone (Toris K) contact lenses (SwissLens, Prilly, Switzerland), which consist of a silicone hydrogel material (filcon V3, definite 74/lgel 77), are specially designed for patients with irregular corneas, or keratoconus^(18-23)^. Toris K lenses have a front toric surface with additional spheric and cylindrical corrections and bumps at 0° and 180°, which provide dynamic stabilization for the lens. Although these soft contact lenses are designed for keratoconus, they are reported to be effective in patients with steep and irregular corneas due to corneal scars or surgery such as penetrating keratoplasty for the management of keratoconus^(20,22,23)^.

These studies have suggested the use of such lenses in patients with PM who have steep corneas. Thus, the aim of this study was to evaluate the efficacy of soft HydroCone silicone hydrogel contact lenses in patients with PM who have higher hyperopia and steeper cornea than the normal population.

## METHODS

Twenty six eyes of 13 patients who were admitted with unwillingness to use spectacles, had discomfort with rigid gas-permeable lenses, and were diagnosed as having PM were fitted with soft HydroCone silicone hydrogel contact lenses (Toris K). The patients were recruited from the contact lens department between 2016 and 2018, and their charts were reviewed retrospectively. The mean ± SD age of the patients was 24.22 ± 7.39 years (range, 9-36 years). None of the patients had a history of consanguinity or any associated systemic disorder. Informed consent was obtained from all participants and from a parent and/or legal guardian of participants aged <18 years in accordance with the principles stipulated in the Declaration of Helsinki. The study was approved by the ethics committee of the Turkish Ministry of Health, Taksim Education and Re­search Hospital, Istanbul, Turkey. The exclusion criteria consisted of histories of any ocular surgery, severe dry eye, active keratitis, and trauma. To be included in this study, each patient must meet all the criteria indicated in previous studies on PM as follows^(11)^: hyperopia ≥8 diopters (D) with cycloplegic refraction, normal-subnormal anterior chamber dimensions and axial length ≤20 mm, and inability to fit with standard soft contact lenses.

All the patients underwent complete ophthalmic examination, including uncorrected and best-corrected visual acuity, using the logarithm of the minimum angle of resolution (logMAR) chart (Smart System II 2020 Visual Acuity System; M&S Technologies, Inc, Skokie, IL) and slit-lamp examination for anterior segment and intraocular pressure measurement. After cycloplegic refraction was obtained with an autorefractor-keratometer (Canon-RK F1, Canon Medical Systems, Ontario, Canada), non-contact lens fundus examination was performed. Standardized optical biometry (IOL Master; Carl Zeiss Meditec AG, Jena, Germany) was performed to measure the axial length, central anterior chamber depth, lens thickness, length of the vitreous cavity, and keratometry readings of the steep and flat corneal meridians. The corneal topography was analyzed with the Sirius System (CSO, Florence, Italy), which provided information about the anterior and posterior curvatures of the cornea, using parameters, including corneal astigmatism, axis of astigmatism, mean keratometric values, central corneal thickness, and horizontal visible iris diameters before and after lens fitting.

The lenses were chosen according to the parameter values obtained from the topographical analysis, from the trial set that included lenses with different base curves and peripheral radii (Table 1)^(19)^. All the patients were fitted with Toris K contact lenses in accordance with the manufacturer’s specifications, as indicated in the technical guides (Table 2)^(19-21)^. After fitting the lenses and being satisfied with the patients’ comfort, the residual refractive power was measured with an automated refractometer, and overrefraction was performed. After 30 minutes of waiting, dynamic stabilization marks were evaluated, and a push-up test was performed. After the patients were examined for perfect fitting, including movement, rotation, centralization, comfort, and bulbar hyperemia or blanching, the best contact lens-corrected visual acuity was measured.

**Table 1:** Fitting set parameters of Toris K

Technical data	Value
Total diameter	13.70 mm (HydroCone K12)
	14.00 mm (HydroCone K34)
Base curve	7.20 to 8.40 D
Sphere	-40.0 to 40.0 D
Cylinder	-0.01 to-8.00 D
Axis	0-180
Center thickness	Standard K12 = 0.42 mm, K34 = 0.52 mm
	Range of thickness: 0.352-0.59 mm
Flattening	HydroCone K12+
	HydroCone K34++

**Table 2:** Fitting assessment procedure of soft HydroCone (Toris K) lens

First contact lens choice
Working with trial lenses with a cylindrical power of-0.01 D is suggested
Keratoconus classification
First, apply topographical indications, or follow the rules:
Vcc >0.6 and/or keratometry >6.8: grade 1 or 2 (choose HydroCone K12)
Vcc <0.6 and/or keratometry <6.8: grade 3 or 4 (choose HydroCone K34)
Diameter and base curve selection
Add 0.8 D to the average K value, and then select a trial lens
HydroCone K12/total diameter = 14.00 mm
HydroCone K34/total diameter = 13.70 mm

All statistical analyses were performed using SPSS version 22.0 for Windows (SPSS Inc., Chicago, IL, USA). Descriptive statistics included mean ± SD for normally distributed variables and were analyzed for demographic data, refractive status, and topographical and contact lens parameters. A paired-samples *t* test was used to compare the statistical significance of the change in visual acuity and other parameters. A p value <0.05 was considered statistically significant.

## RESULTS

In our patients, cycloplegic refraction showed hyperopia ranging from 10 to 21.5 D. The spherical equivalent of the right eye (oculus dexter [OD]) ranged from 10.00 to 19.25 D, and that of the left eye (oculus sinister [OS]) ranged from 11.00 to 21.5 D (mean ± SD, 14.61 ± 2.63 D and 15.38 ± 2.99 D, respectively). Intraocular pressure measurements were normal in all the patients. Dilated fundus examination revealed a crowded optic disc appearance in all the patients and papillomacular folds in 10 eyes. Data obtained from the measurements of the two eyes of a given individual were similar and are summarized in table 3.

**Table 3:** Biometric analysis for patients with posterior microphthalmos

	Right eye	Left eye
Axial length (mm)	15.99 ± 1.00	15.92 ± 1.09
Horizontal visible iris diameter (mm)	11.88 ± 0.74	11.85 ± 0.68
Anterior chamber depth (mm)	3.01 ± 0.30	2.99 ± 0.33
Lens thickness (mm)	3.96 ± 0.54	3.88 ± 0.98
Posterior chamber length (mm)	7.19 ± 1.12	8.1 ± 0.92
Spheric equivalent (D)	15.02 ± 2.8	15.80 ± 3.11
K1 (D)	48.58 ± 1.79	48.60 ± 1.50
K2 (D)	49.79 ± 1.49	49.77 ± 1.46
Mean *Kmax (D)*	49.18 ± 1.74	49.25 ± 1.55

The mean axial lengths for all the patients were 15.99 ± 1.00 mm OD and 15.92 ± 1.09 mm OS, and the mean posterior chamber lengths were 7.19 ± 1.12 mm OD and 8.1 ± 0.92 mm OS, both shorter than those of the age-matched population^(18)^. The mean horizontal visible iris diameters were 11.88 ± 0.74 mm OD and 11.85 ± 0.68 mm OS (range, 11.22-13.35 mm), mean central anterior chamber depths were 3.01 ± 0.30 mm OD and 2.99 ± 0.33 mm OS (range, 2.70-3.72 mm), and mean lens thicknesses were 3.96 ± 0.54 mm OD and 3.88 ± 0.98 mm OS (range, 3.68-4.11 mm), which are all within the normal ranges^(8,24)^. The mean central corneal thicknesses were 556 ± 21 µm OD and 541 ± 15 µm OS, which are also within the normal range^(25)^.

**Figure 1:**
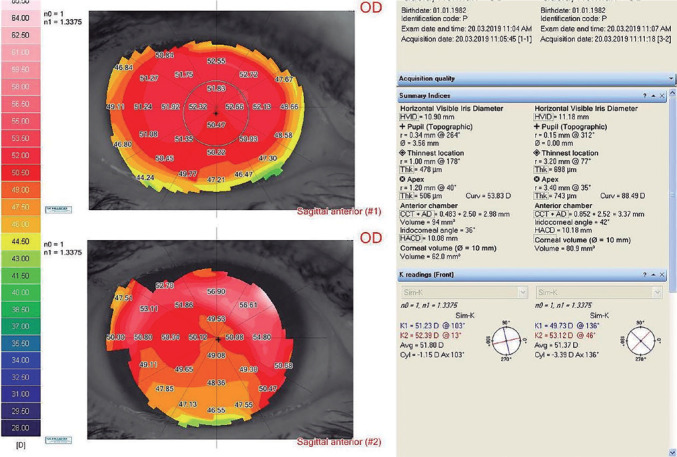
Corneal topography in a patient before and after Toris K lens fitting.

The keratometry measurements obtained from the corneal topography revealed increased corneal curvature, with a mean K1 of 48.59 ± 1.61 D (48.58 ± 1.79 OD and 48.60 ± 1.50 OS), mean K2 of 49.78 ± 1.43 D (49.79 ± 1.49 OD and 49.77 ± 1.46 OS), and mean *K*_max_ of 49.22 ± 1.59 D (49.18 ± 1.74 OD and 49.25 ± 1.55 OS), which were steeper than in the normal po­pulation^(26)^ (Figure 1).

All the patients were fitted with HydroCone K34 in accordance with the manufacturer’s indications as shown in table 2. The preferred diameter for perfect fitting in the microphthalmic corneas was 13.3 mm. The power of the lenses ranged from 8 to 17 D (median, 13 D) The base curves of the lenses ranged from 7.30 to 7.80 (median, 7.50).

The mean logMAR visual acuity was 0.61 ± 0.47 with spectacles (0.70 ± 0.48 OD and 0.52 ± 0.46 OS) and 0.45 ± 0.24 with contact lenses (0.48 ± 0.26 OD and 0.42 ± 0.23 OS). The mean best contact lens-corrected visual acuity was significantly higher than the mean best spectacle-corrected visual acuity for both eyes (p=0.042 and p=0.032, respectively; paired-samples *t* test; Table 4). The mean follow-up time was 2 years. Eight of the 13 patients stopped using contact lenses after 1 year because of the high cost and continued with the use of spectacles. Five patients continued to wear their lenses with annual replacements.

**Table 4:** Comparison of best-corrected visual acuity between soft HydroCone (Toris K) lenses and spectacles

	Best spectacle-corrected visual acuity (logMAR)	Best contact lens-corrected visual acuity (logMAR)	P value
Right eye	0.70 ± 0.48	0.48 ± 0.26	0.042*
Left eye	0.52 ± 0.46	0.42 ± 0.23	0.032*

* Paired-samples *t* test

Only one patient had conjunctival hyperemia in both eyes, which lasted for 2 days, and two patients had superficial punctate keratitis, which was treated with artificial tear substitutes.

## DISCUSSION

The causes of low visual acuity in patients with PM are high refractive error (ametropia), posterior segment abnormalities, and amblyopia^(27)^. Ametropia has been divided into two categories according to the three variables of refraction, namely axial length, corneal shape, and lens power^(25,28)^. Correlation ametropia is described as inappropriate correlations of these three variables within the mean normal ranges, resulting in refraction ranging from-4 to 6 D. Component ametropia is described as one or more of these variables deviating from mean values, where the deviation of these variables results in refraction outside the range of-4 to 6 D. Although anterior segment structures are normal in size, the posterior segment is shorter than normal in PM. Owing to disparity due to arrested development of the sclera, this disorder can be grouped under the heading of component ametropia. Corneal steepening has been reported in patients with PM^(4,7,9,11,16,17)^. This might be due to the emmetropization process to balance the axial length of the eye and the refraction of the anterior segment to achieve near-normal refraction where corneal curvature and refractive power increase to compensate for the short axial length. Nowilaty et al.^(11)^ described the biometric characteristics of 25 patients clinically diagnosed as having PM from 13 families. They reported the biometric similarity of the two eyes of a given individual, with an average spheric equivalent of +15.09 D and a mean axial length of 16.25 mm. The central anterior chamber depth and axial lens thickness were within the normal ranges. However, the average corneal power was 48.89 D, which indicated steepening. They found that total axial length negatively correlated with average corneal power and that the degree of hyperopia and positively correlated with horizontal corneal diameter and axial vitreous length. Relhan et al.^(16)^ also analyzed the biometric differentiation of PM from nanophthalmos. They reported better visual acuity than those of patients with nanophthalmos who had increased keratometric values (mean, 46.01 D) and high hyperopia (11.59 ± 3.28). In the present study, increased keratometric values were detected in the patients with PM, indicating a steeper cornea. In this study, the anterior segment values, including central anterior chamber depth, axial lens thickness, horizontal visible iris diameter, and central corneal thickness, were within the normal ranges, similar to those reported in previous studies^(8,11,24,25)^.

Correction of high hyperopia with spectacles is the primary approach. Despite the availability of specially designed high index lens materials and aspheric lens designs for spectacles, lens thickness, increased weight due to high plus-power lenses, aniseikonia, and magnification are some of the disadvantages of spectacles for patients with significant hyperopia^(29)^. Soft or rigid contact lenses provide some advantages over spectacles, as they improve cosmesis and binocularity and decrease accommodative and convergence demands in hyperopic patients. However, high costs and ocular complications due to corneal hypoxia or infection are the disadvantages of contact lenses. Pradhan et al presented a case of bilateral nanophthalmos in a patient fitted with rigid gas-permeable contact lenses and achieved better visual acuity than spectacle correction^(30)^. Owing to the disadvantages of rigid gas-permeable contact lenses such as intolerance and ocular discomfort in long-term wear as a result of the rigid material of the lens and potential damage to the corneal surface, especially at the apical area, soft contact lenses might be a better alternative for patients with PM who have steep corneas and significant hyperopia.

HydroCone (Toris K) soft contact lenses are custom-made lenses especially designed for keratoconus that have additional spherical and cylindrical corrections to the toric surface of the lens to increase visual performance^(18,19)^. The dynamic stabilization with nasal and temporal bumps and the unique design of these lenses provide oxygen supply at the limbus similar to that from the standard prismatic contact lenses, comfort similar to that from the standard soft contact lenses, and visual acuity similar to that from rigid gas-permeable contact lenses. Previous studies demonstrated that Toris K soft contact lenses offered better best-corrected visual acuity than spectacles for the management of keratoconus^(18,20-23)^. Gumus and Kahraman^(18)^ reported an increase of 4.5 lines in best-corrected visual acuity and significant decrease in mean K1, K2, and *K*_max_ values and baseline topographical spherical equivalent.

**Figure 2:**
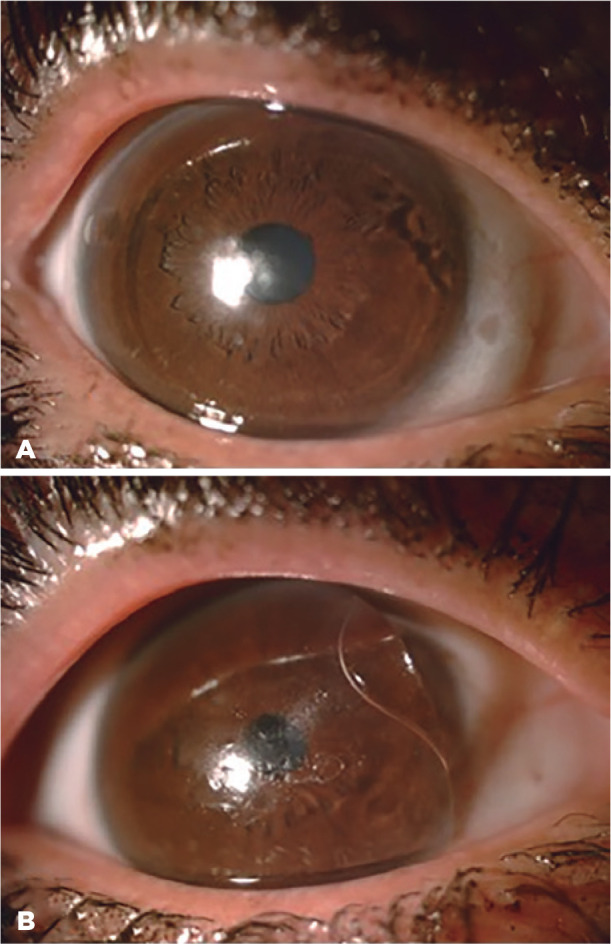
Fitting of the same eye in a patient with a specially designed Toris K lens with nasal and temporal bumps for stabilization (A) and with the standard soft contact lens (B).

The significant improvement in best-corrected visual acuity with Toris K soft contact lenses in the patients with keratoconus who had steep corneas gave us the idea to use these contact lenses in patients with PM with established steep corneas and hyperopia. The unwillingness of patients to use spectacles and inability to fit patients with conventional soft or rigid gas-permeable contact lenses were the reasons for choosing HydroCone (Toris K) soft contact lenses in this study (Figure 2). HydroCone (Toris K) soft contact lenses were preferred, and the visual performances of these lenses were compared with the uncorrected and best spectacle-corrected visual acuities of the patients. Similar to those reported in previous studies, the best-corrected visual acuity with Toris K lenses was significantly better than the uncorrected and best spectacle-corrected visual acuities in this study.

Superficial punctate keratitis, giant papillary conjunctivitis, corneal edema, sterile corneal ulcer, and broken/torn contact lens are the complications reported in the literature^(20)^.

Superficial punctate keratitis in both eyes of two patients and transient conjunctival hyperemia in both eyes of one patient were the complications found with the use of Toris K in this study. These were minor complications and were managed with medical treatment.

The limitation of this study was the small sample size because of the rarity of the disease. To the best of our knowledge, this is the first study to evaluate the efficacy of HydroCone (Toris K) silicon soft contact lenses in patients with PM who had steep corneas.

In conclusion, HydroCone (Toris K) silicon soft contact lenses offer better visual acuity than spectacles in patients with PM. Further studies with a larger number of participants are necessary to establish the efficacy of these specially designed soft contact lenses in patients with PM.
